# A Novel Nontraumatic Gingival Retraction Method for a Single-Unit Crown Impression

**DOI:** 10.1155/2022/3066712

**Published:** 2022-05-18

**Authors:** Hozaifa Al-Nasser, Nabil Al-Houri, Anas Mouti, Heba Alajami

**Affiliations:** Department of Prosthodontics, Faculty of Dental Medicine, Damascus University, Syria

## Abstract

Until now, there have been three traditional options for gingival retraction procedures, mechanical, chemicomechanical, and surgical methods, which have a degree of trauma that varies according to the clinical experience. The gingival tissue may be very delicate and susceptible to recession if too much trauma was applied. This article describes a novel nontraumatic gingival retraction method, without cords, chemicals, surgery, or any special equipment.

## 1. Introduction

Successful prosthesis depends mainly on the preciseness of the dental impression. Thus, it is necessary to choose the appropriate impression technique to achieve accurate dies. There are several impression techniques, and each one has its advantages and disadvantages. The 100% accurate technique does not exist so far [1].

Dentists sometimes face a lot of difficulties in choosing the appropriate impression material and technique. Taking into consideration the ease, speed of work, and patient satisfaction. There are many difficulties in achieving accurate impressions for multiprepared teeth, especially in the case of subgingival finish line [2].

To reach a good and precise impression, 0.2 mm of gingival retraction is enough [3], while 0.15–0.2 mm under the finish line is preferable [4]. Another study concluded that we need at least 0.2 to 0.3 beneath the finish line [5].

Any technique of gingival retraction needs a dry field and sufficient retraction of the gingival tissues to facilitate the flow of the impression material without harming the gingiva.

The current methods of gingival displacement are divided into mechanical, chemicomechanical, and surgical methods [6].

Mechanical manners are achieved effectively by the placement of a cord (generally impregnated with a medicament) [7]. It is a cheap manner, but it may traumatize epithelial attachment due to its lacking of tactile sensation. Also, there is a chance of subsequent bleeding after removing the retraction cord, which occurs in 50% of the cases [6]. The copper ring was used in the past, but it may hurt the gingiva during removal, especially in the case of undercut presence [8]. The chemical agents that are commonly used with retraction cords are listed in (Table 1) [9].

Foam or paste systems with direct pressure are considered cordless methods. While surgical tissue removal can be accomplished through rotary curettage, excision with a scalpel, electrosurgery [7], or laser, which has minimum postoperative pain and discomfort [6], it is important to select the best method of gingival displacement to have an accurate impression without harming the gingiva and according to the patient's health condition at the same time.

This paper is aimed at demonstrating a tip for clinicians in retracting the delicate gingiva, especially in anticoagulants patients, in a cost-effective and time-preserving way, eliminating the need for high clinical skills.

## 2. Clinical Report

A 55-year-old woman referred to the prosthodontics department, faculty of dental medicine, Damascus University, to restore her maxillary endo-treated molar.

Asymptomatic abutment tooth with an adequate amount of tooth structure was observed on clinical assessment. An intraoral periapical radiograph revealed a good root filling without any periapical radiolucency and complete obturation of all the canals.

The medical history of the patient revealed that she has taken anticoagulants, conjugated with hyperthyroidism.

All prosthetic options were discussed with the patient in great detail. Porcelain fused to metal crown (PFM) was chosen.

**Figure 1:**
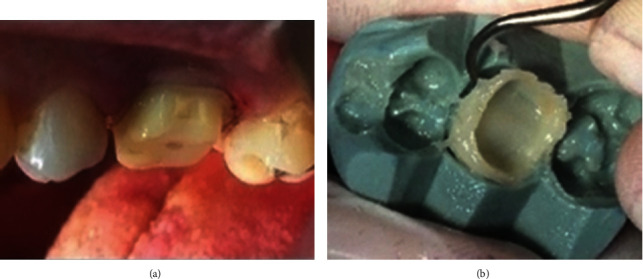
(a) The prepared abutment. (b) The temporary crown before trimming.

**Figure 2:**
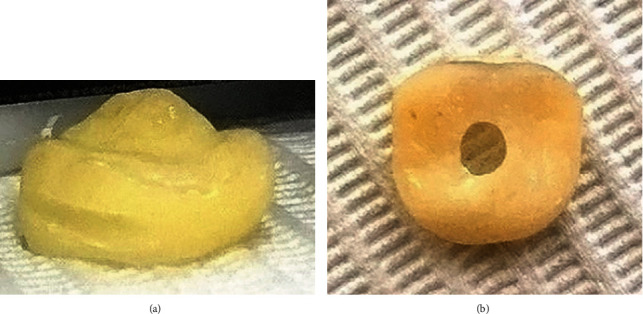
(a) The horizontal groove. (b) Hole for excess impression material drainage.

**Figure 3:**
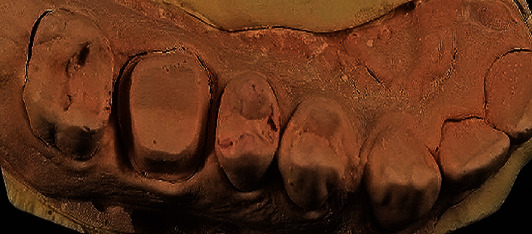
The final cast.

The following step-by-step procedure was performed:

1. The provisional (temporary) crown was fabricated using a silicon index (Zeta Plus, Zhermak Spa, Italy) for the abutment before it was prepared
2. After the abutment has been prepared, cold-cured acrylic resin (R-dental Dentalerzeugnisse GmbH, Germany) was injected into the silicon index and pressed firmly on the abutment. After the material had set, the crown was removed and trimmed using a carbide bur (Maxi-Cut; Lesfils de August Malleifer SA, Ballaigues, Switzerland) (Figure 1).
3. A horizontal groove was drilled on the buccal surface of the temporary crown as a guiding groove, which facilitated its placement over the prepared tooth and as an undercut for fixing the impression material (Figure 2(a))
4. A hole was drilled on the occlusal surface of the crown to facilitate the flowing of the excess light polyvinyl siloxane (Figure 2(b)).

(5) The temporary crown was filled with light body silicone and pressed on the prepared tooth with a finger to adapt firmly
(6) A pick-up impression was made using putty silicone
(7) The impression was poured with dental stone type III to obtain the final cast (Figure 3).

## 3. Discussion

Managing the gingival tissue for impression making is a challenging aspect of fixed prosthodontics, especially when the gingiva is fragile and susceptible to bleeding, such as the patient in this report, whom she has taken anticoagulants and has hyperthyroidism, which made the use of retraction cords contraindicated.

In general, an accurate impression demands capturing the margins of the prepared abutment with minimal traumatization to the surrounding tissues. We can notice that all retraction methods have a degree of trauma. In addition to the fact being a patient-annoying and time-consuming procedure. On the other hand, some kinds of chemicals used in some methods may damage the surrounding tissues [10].

In 1965, a new impression technique was found depending on the direct intraoral provisional prosthesis, using a wax matrix. This method has limited the need for a gingival retraction but the material used as a matrix was not accurate [11].

**Figure 4:**
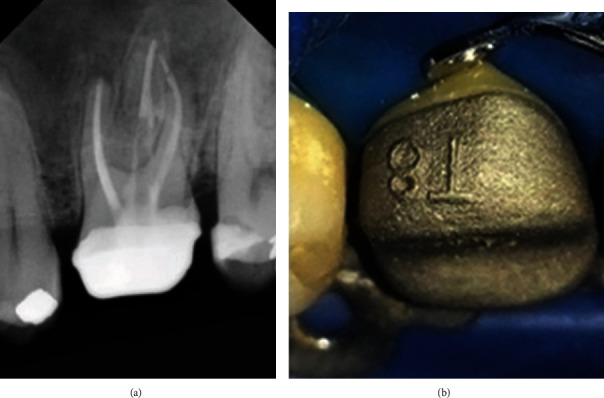
(a, b) Marginal fit of the final crown.

An accurate elastomeric impression is achieved using a custom tray as it decreases the volume of the material and reduces in turn the stresses during impression removal and the subsequent thermal contraction [7]. Therefore, the Provisional Crown-Impression technique was developed using a silicon matrix to have an accurate impression as much as possible. We found that this manner has produced a satisfactory marginal fit clinically and radiographically as demonstrated in Figure 4.

Previous studies revealed that the accuracy of the impression was affected by the thickness of the impression material, even in relatively stable materials [12]. Thus, using a temporary crown decreases the bulk of the elastomeric impression material and reduces the polymerization shrinkage of the light body silicone, which has a low amount of filler [13].

The gingiva was mechanically retracted by applying pressure, and thus, the impression material was pushed into the gingival sulcus. This technique was used as a conservative and painless process, by eliminating the use of cords or any other invasive method. Hence, it reduced the risk of irreversible gingival recession after the impression.

We found that the Temporary Crown-Impression technique was time-preservative as it was performed in one stage, in comparison with the two stage-putty wash technique, which needs additional time for local anaesthesia and packing the cord, especially if we have multiple abutments.

In the present paper, we used Zeta Plus impression material with 30 s for mixing time, 75 s for working time, and 195 s to get set. As for the temporary crown, it took about 180 s to be fabricated and no more than 330 s for making the one-stage impression.

To compare manual and digital impressions, the latter has some advantages over the manual one. It reduces the patient's pain and discomfort compared with the manual impression where the agar or elastomeric materials are placed directly in the patient's mouth. The intraoral scanner is more suitable for elders and patients with a strong gag reflex. It reduces the risk of infection, the cost, and materials wasting. It can be shared as three-dimensional data transmitted through the network. In contrast, the digital impression has some limitations like training requirements and visualization of a dry field in addition to the scanning fees [14].

## 4. Conclusion

Compared to all available retraction procedures, the Provisional-Crown Impression technique may be considered as an atraumatic and cost-effective alternative way, especially in case of failure to obtain an accurate impression by the traditional means, without the need for professional skills or high technical sensitivity. It may aid in controlling fluids and bleeding, which was an advantage in the case of multiple prepared abutments. It was also useful in case of having any contraindication to various methods of gingival displacement.

## Figures and Tables

**Table 1:** Chemical agents used with retraction cords [9].

Chemical agent	Effect
Epinephrine	Despite the effectiveness of epinephrine as a vasoconstrictor, there is a great possibility of having an overdose, whereas its maximum recommended dose for a cardiovascular patient is 0.04 mg and each impregnated thread contains 0.2-1 mg of epinephrine, which may cause serious side effects, especially if the retraction procedure is performed after local anaesthesia combined with adrenaline
Ferrous sulfate	Although it has a hemostatic effect, it causes irritation and tissue staining.
Zinc chloride	It is used rarely as it reveals a caustic effect.
Aluminium-based agents	Although their safety and absence of systemic effects, they became toxic in concentrations of more than 10%
